# Immortalized erythroid cells as a novel frontier for in vitro blood production: current approaches and potential clinical application

**DOI:** 10.1186/s13287-023-03367-8

**Published:** 2023-05-24

**Authors:** Christian Felice Cervellera, Chiara Mazziotta, Giulia Di Mauro, Maria Rosa Iaquinta, Elisa Mazzoni, Elena Torreggiani, Mauro Tognon, Fernanda Martini, John Charles Rotondo

**Affiliations:** 1 Department of Medical Sciences, University of Ferrara, 64/b, Fossato di Mortara Street, 44121 Ferrara, Italy; 2 Department of Medical Sciences, Center for Studies on Gender Medicine, University of Ferrara, 64/b, Fossato di Mortara Street, 44121 Ferrara, Italy; 3 Department of Chemical, Pharmaceutical and Agricultural Sciences—DOCPAS, University of Ferrara, 44121 Ferrara, Italy; 4 Laboratory for Technologies of Advanced Therapies (LTTA), University of Ferrara, 44121 Ferrara, Italy

**Keywords:** Erythroblasts, Immortalization, Hematopoietic stem cells, Erythropoiesis, Cell differentiation, Genetic engineering

## Abstract

**Background:**

Blood transfusions represent common medical procedures, which provide essential supportive therapy. However, these procedures are notoriously expensive for healthcare services and not without risk. The potential threat of transfusion-related complications, such as the development of pathogenic infections and the occurring of alloimmunization events, alongside the donor’s dependence, strongly limits the availability of transfusion units and represents significant concerns in transfusion medicine. Moreover, a further increase in the demand for donated blood and blood transfusion, combined with a reduction in blood donors, is expected as a consequence of the decrease in birth rates and increase in life expectancy in industrialized countries.

**Main body:**

An emerging and alternative strategy preferred over blood transfusion is the in vitro production of blood cells from immortalized erythroid cells. The high survival capacity alongside the stable and longest proliferation time of immortalized erythroid cells could allow the generation of a large number of cells over time, which are able to differentiate into blood cells. However, a large-scale, cost-effective production of blood cells is not yet a routine clinical procedure, as being dependent on the optimization of culture conditions of immortalized erythroid cells.

**Conclusion:**

In our review, we provide an overview of the most recent erythroid cell immortalization approaches, while also describing and discussing related advancements of establishing immortalized erythroid cell lines.

## Introduction

Blood transfusion is an essential clinical procedure, which restores the oxygen transport capacity of circulating blood and consequently improves the amount of circulating hemoglobin and iron. The continuous supply of blood is essential for patients suffering from a wide range of diseases and clinical conditions. Elective surgical procedures, chemotherapy the treatment of chronic hematological conditions, such as anemia, hemophilia, thalassemia and sickle cell anemia, require a continuous and/or significant transfusion of red blood cells [1, 2]. Moreover, blood transfusions undoubtedly provide essential supportive therapy for transfusion needs arising from trauma and acute blood loss. According to the World Health Organization (WHO), blood transfusion is considered the most applied cellular therapy, with roughly 118 million transfusion units administered globally/year [3]. Blood transfusions are known to be expensive due to both storage and processing procedures, while hospital, personnel and equipment fees represent an additional important cost for the healthcare services. Therefore, blood transfusions are considered as a significant burden for both the hospital management systems and the country’s economies [4]. Moreover, all industrialized countries are experiencing important demographic changes as a consequence of the decrease in birth rates and increase in aging demographic of the baby boom generation [5]. An increase in the demand of donated blood, paralleled with reduction in blood donors, is therefore expected [5].

The safety of blood transfusion has increased over the last years, until currently being considered as an essential supportive therapy. However, this procedure presents several risks that clinicians need to be able to deal with. Individuals/patients receiving transfused blood can potentially be exposed to adverse events including pathogenic infections and the occurring of alloimmunization events, some of which could be seriously life-threatening [6]. The presence of bacteria, viruses and protozoa from asymptomatic donors represents a significant route of transmission through blood. The transmission of harmful bacteria via contaminated blood is currently considered major sources of morbidity and mortality in the USA [7, 8]. Likewise, given the possible presence of viruses in blood cells or plasma of healthy blood donors [9], viral infections are considered a key threat in the transfusion medicine, with serious health consequences [10–12]. Infectious agents/diseases, which are transmissible by blood transfusions, are known as transfusion-transmitted diseases and mainly comprise parasitic, viral and prion diseases [13]. Alloimmunization provides the exposition of foreign red cell antigens and the consequential development of immune responses, as result of blood transfusions. In certain circumstances, alloantibodies can be generated following ~ 5% of all blood transfusions, leading to reactions. Additional complications associated with blood transfusions comprise non-infectious/non-immunologic hazards [14].

Active pre-clinical research is ongoing to identify and develop reliable alternative procedures to blood transfusions [15]. An emerging alternative strategy over blood transfusion is the in vitro production of blood cells from immortalized erythroid cells. The highest survival capacity alongside the stable and longest proliferation time of immortalized erythroid cells could allow the generation of a large number of cells, which are able to differentiate into red blood cells [16]. However, it should be noted that the large-scale, cost-effective production of red blood cells is not yet a routine procedure in clinical practice due to the still ongoing optimization of culture conditions of immortalized erythroid cells. This review provides an overview on the most recent erythroid cell immortalization approaches, while discussing related advancements in establishing immortalized erythroid cell lines.

### Erythropoiesis

Erythropoiesis is a complex process that leads to the production of mature, enucleated red blood cells (RBCs). In healthy adults, about 2 million RBCs are produced every second in the bone marrow and are released into the peripheral blood. RBCs ensure appropriate oxygen amounts supplied to peripheral tissues, while in turn keeping stable hemoglobin concentrations [17]. Two distinct RBC populations named embryonic and adult have been described. Both populations arise from distinct hematopoietic progenitors in different anatomical sites and show specific genetic programs [18]. Primitive RBCs are the first blood cells generated during embryonic hematopoiesis. They consist of large and nucleated erythroid cells that originate from the yolk sac and support the growth and survival of the embryo/fetus [19]. Definitive RBCs consist of small enucleated blood cells, arising from the fetal liver and postnatal bone marrow, and provide erythroid cell production not only during late fetal life but also throughout the postnatal life.

**Fig. 1:**
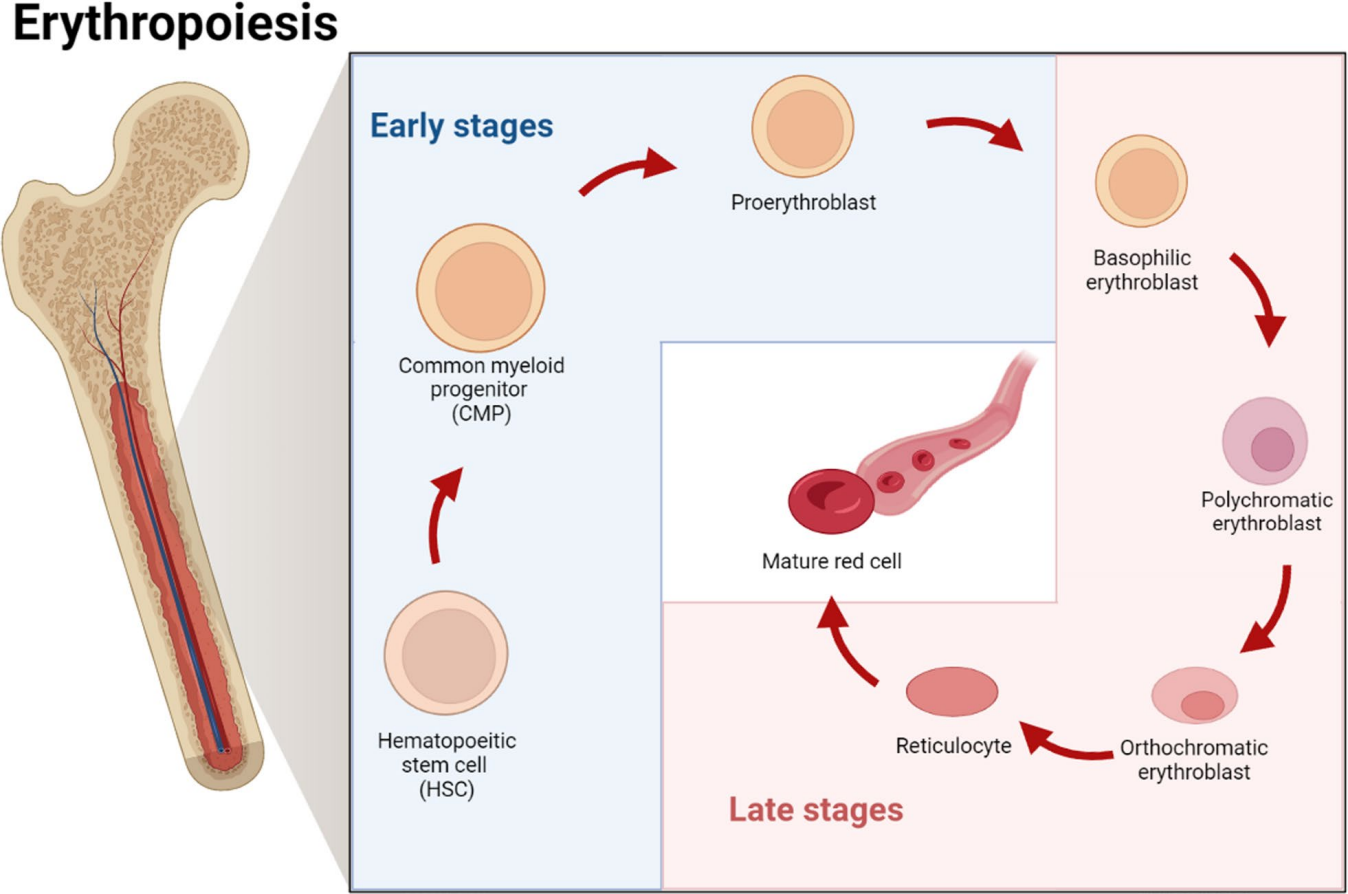
Overview of the erythropoiesis process which provides the production of mature red blood cells. Erythropoiesis can be divided into two stages of differentiation. In the first, known as early erythroid differentiation stage, hematopoietic stem cells (HSCs), located in the bone marrow, differentiate first into common myeloid progenitors and then into proerythroblasts. In the second, known as late erythroid differentiation stage, proerythroblasts become in sequence basophilic, polychromatic and orthochromatic erythroblasts, upon erythropoietin stimulation. Upon expelling nuclei and losing all organelles, orthochromatic erythroblasts become reticulocytes. Lastly, reticulocytes are then released into the circulation and begin their maturation to become functional red blood cells. Figure was made by the authors of this review by using BioRender online tool (www.biorender.com). Figure is not under copyright

Definitive erythropoiesis, in the adult organism, is characterized by the movement of lineage-committed cells through progenitor, precursor and mature RBC compartments, over two major phases (Fig. 1) [20]. The earlier phase originates with multipotential hematopoietic stem and progenitor cells (HSPCs) that give rise to erythroid-committed progenitors. The latter phase leads to the maturation of erythroid precursors into enucleated reticulocytes, which then undergo terminal maturation into RBCs in the bloodstream. In the early phase, multipotential HSPCs, named hemocytoblasts, are able to differentiate either (1) through a common myeloid progenitor (CMP) intermediate, which is able to mature into a megakaryocyte–erythroid progenitor or (2) directly into a more committed erythroid progenitor, which will soon mature into the first erythroid precursor, named proerythroblast. Lastly, the maturation process leads to the burst-forming unit erythroid (BFU-E) that can form colony-forming unit erythroid (CFU-E) [18, 21, 22]. These progenitors are defined by their ability to form colonies of mature erythroid cells in semisolid media. Specifically, BFU-E is able to form mature colonies of thousands erythroid cells in approximately 14 days. In contrast, the more mature CFU-E progenitors give rise to mature colonies consisting of only 16–32 cells in 7 days [23, 24]. Subsequently, CFU-Es undergo a series of maturation steps collectively named terminal or late erythropoiesis, which consists of morphologically identifiable, nucleated precursors. This erythroid maturation phase starts from the most immature erythroid precursors, named proerythroblasts and progresses to basophilic, polychromatophilic and orthochromatic forms. The terminal erythropoiesis is characterized by erythroblast expansion, gradual accumulation of hemoglobin, decrease in cell size and progressive nuclear pyknosis, ultimately resulting in enucleation [25]. Erythroid precursor maturation occurs in the bone marrow, within erythroblastic islands, specific anatomic niches composed of a central macrophage surrounded by up to 30 erythroblasts at various degrees of maturation [26]. In this site, macrophages bund to erythroblasts within the islands, providing them iron and the cellular interactions necessary to promote erythroid proliferation and maturation [27]. The final step of precursor maturation consists of the loss of cytoplasmic organelles, such as mitochondria, ribosomes, the Golgi apparatus and the endoplasmic reticulum, as well as nucleus expulsion through enucleation [28]. Enucleation is a three-step process characterized by drastic molecular and cellular changes including (1) cell cycle arrest, (2) nuclear polarity establishment and (3) chromatin and nuclear condensation [29, 30]. In particular, the nuclear polarization is driven by rearrangements of actin cytoskeleton and the clathrin-dependent generation of vacuoles at the nuclear–cytoplasmic junction [31]. Nuclear and chromatin condensation is dependent on the acetylation status of histones H3 and H4 under the control of histone acetyl transferases and histone deacetylases [32, 33], which have been shown to be essential for the formation of the contractile actin ring, implicated in nuclear pyknosis [34]. Overall, enucleation results in the formation of two cell populations, i.e., reticulocytes and pyrenocytes. The former is characterized by most of the cytoplasm and hemoglobin, and proteins needed to form a unique cytoskeletal network [35]. The latter contains condensed nucleus surrounded by a lipid bilayer and tiny cytoplasm layer [36]. Soon after their formation, pyrenocytes transfer phosphatidylserine onto their cell surface providing a so-called eat-me signal that prompts macrophages of the erythroblastic island to rapidly eliminate them [37]. Conversely, after expelling their nucleus, reticulocytes continue to mature. Reticulocyte maturation results in (1) 20–30% loss of plasma membrane surface [38]; (2) increased association of the cytoskeleton to the outer cell membrane; (3) reduced cell volume; and (4) elimination of all remaining cytoplasmic organelles, through both autophagy and exocytosis/exosome-combined pathway [39].

### Stem cell-based approaches to red blood cell production

RBC transfusion is one of the most common medical treatments. Given the imbalance in the RBC supply/demand ratio, especially for alloimmunized patients/individuals or patients with rare blood phenotypes, extensive research has been carried out to generate therapeutic quantities of mature RBCs from hematopoietic stem cells (HSCs) of various sources.

Bone marrow (BM) has been considered the only source of HSCs for more than a decade following the first bone marrow transplantation occurred in the mid-50s [40]. The lack of an acceptable number of HSPCs obtained from BM encouraged additional studies aimed at identifying alternative stem cell sources. Later, preliminary animal-based studies showed the presence of circulating HSPCs in the blood of mice [41]. However, the attempts of peripheral blood hematopoietic stem cell transplantation were mostly ineffective due to the scarcity of stem cells in the peripheral blood [42]. The administration of recombinant growth factor granulocyte colony-stimulating factor (G-CSF) can mobilize HSPCs from the bone marrow and lead to a significant rise in the number of circulating CD34^+^ progenitor cells in the blood [43]. The long-term safety in allogeneic recipients of peripheral blood CD34^+^ HSC might arise potential concerns relying on the high number of donor lymphocyte T cells present in the peripheral blood, alongside the potentially increased risk of graft versus host diseases [42]. However, peripheral blood hematopoietic stem cell transplantations represent standard procedures in clinical practice.

Umbilical cord blood (UCB) is currently recognized as a valuable cell source, while being widely used in HSC transplantations [44]. UCB-HSCs are immediately available and can be collected with no risk to either the mother or the newborn. However, the number of nucleated cells in the UCB unit is ten times smaller than in BM and/or peripheral blood grafts. This reflects clinically into a higher incidence of engraftment failure and longer time to cell recovery. Transplants with UCB result in significantly lower rates of acute/chronic graft versus host diseases [45].

Cellular/gene therapies for hematologic disorders can benefit from the generation of HSPCs or mature blood cells from human pluripotent stem cells (hPSCs). HPSCs, both embryonic (hESCs) and induced PSCs (iPSCs), offer a plentiful source of blood cells for experimentation and therapeutic purposes [46]. The advantage of iPSCs is that (1) their cell source is easily accessible; (2) they can be produced at a relatively reasonable price; and (3) they represent a virtually unlimited source of HSPCs and mature blood cells. The plasticity of human iPSCs with the potential to differentiate into virtually any type of cells and the feasibility of generating HSPCs from patient-derived iPSCs give numerous potential hematological applications for these cells [47]. Indeed, the generation of patient-derived iPSCs and their subsequent differentiation into iPSC-HSPCs offer a unique opportunity for generating disease models to study genetic/immune diseases [47]. The reprogramming of human somatic cells through the ectopic expression of the transcription factors such as OCT4, SOX2, KLF4, c-MYC, LIN28 and NANOG has provided a new avenue for disease modeling and regenerative medicine [48]. Various techniques have been developed to generate enucleated RBCs from human iPSCs [49]. The in vitro production of human iPSC-derived RBCs can be an alternative treatment option for patients with blood disorders [48]. Much attention has focused on human PSCs to replace current transfusion banking. Generating RBCs from gene-edited iPSCs would allow to overcome the occurrence of erythrocyte alloimmunization by recipients of repeated transfusions such as sickle cell anemia and thalassemia patients [50].

Peripheral blood mononuclear cells (PBMCs) represent a valuable alternative source of erythroid cells. PBMCs are a heterogeneous class of mononuclear cells identified as any blood cell with a round nucleus, such as lymphocytes, monocytes, natural killer (NK) cells and/or dendritic cells, being isolated from peripheral blood. Although this source is characterized toward intrinsic DNA rearrangements that may influence cell differentiation in vitro, especially in T/B progenitor cells [51, 52], PBMCs isolation represents a significant and minimally invasive solution for establishing CD34^+^ cultures from healthy blood donors [53, 54].

### Immortalization approaches of erythroid cells

#### Human papillomavirus-based immortalization approaches

The currently developed erythroid cell immortalization approaches mainly comprise HPV-based in vitro systems (Table 1). Oncogenic HPVs, particularly HPV16/18, are associated with onset of genital and oropharyngeal cancers [55–57]. The HPV oncogenicity is related to the activity of viral oncoproteins *E6* and *E7*, which are able to promote cell cycle progression, as well as cell growth and proliferation through p53 and pRB suppression [58]. According to *E6/E7* transforming abilities, exogenous expression of both oncogenes has been largely exploited to perform immortalization approaches of erythroid cells [16, 59–61].

**Table 1:** In vitro generation of immortalized red blood cell line using human papillomavirus oncogenes

References	Vector/plasmid/other systems for immortalization	Genes used for immortalization	Inducible	Cell origin	Cells used	Proliferation time	Hemoglobin production	Percent of enucleation (%)
Akimov et al. [62]	Lentiviral vector	hTERT and/or HPV16 E6/E7	No	UCB	CD34+	~ 24 months	–	–
Wong et al. 2010	Lentiviral vector	SV40T, hTERT, or HPV16 E6/E7	No	PB	CD36+	> 1 month	*γ*- > *β*-globin	–
Kurita et al. [59]	Lentiviral vector	HPV16 E6/E7	Tet-inducible	UCB	iPS cell lines and CD34+	> 12 months	*β*- or *γ*-globin	N.R
Trakarnsanga et al. [60]	Lentiviral vector	HPV16 E6/E7	Tet-inducible	BM	CD34+	> 6 months	~ 99% *β*-globin	30
Kurita et al. [61]	Lentiviral vector	HPV16 E6/E7	Tet-inducible	BM	CD34+ and CD34+	from 1 to 8 months	~ 96% *β*-globin ~ 2% *γ*-globin	~ 25
Trakarnsanga et al. [69]	Lentiviral vector	HPV16 E6/E7	Tet-inducible	*β*-thalassemic PB patient	CD34+	> 3 months	29.2% of HbF 9.0% of HbA 61.8% of HbE	~ 10
Daniels et al. [16]	Lentiviral vector	HPV16 E6/E7	Tet-inducible	BM, PB, UCB	CD34+	> 6 months	BEL-C: *γ* > *β* BEL-P: *β-* > *γ*-globin	~ 26
Bagchi et al. [76]	Lentiviral vector	HPV16 E6/E7	No	PB BM	PB-EPCs CD34+	3–4 months	PBiEPC: *α*- and *β*-globins CD34 + iEPC: *γ*-globin	From 2 to ~21
Soboleva et al. [75]	Lentiviral vector	HPV16 E6/E7	No	BM	CD34+	> 11 months	*β-* > *γ*-globin	N.R

BM, bone marrow; EPCs, erythroid progenitor cells; N.R., not reported but evaluated; PB, peripheral blood; UCB, umbilical cord blood; –, not reported and not evaluated

One of the first attempts to immortalize an erythroid lineage with a viral vector has been performed by Akimov et al*.*, generating a cord blood CD34^+^ immortalized cell line through the lentiviral co-transduction of HPV16 E6 and E7 and human telomerase reverse transcriptase (hTERT). The established cell line was able to extend the normal lifespan of myeloerythroid/mast cell progenitors and did not display a tumorigenic activity, although abnormal karyotypes/chromosomal changes were described [62].

Kurita et al*.* took an important early step into the RBCs in vitro immortalization understanding [59]. Upon the generation of immortalized mouse embryonic stem cell-derived erythroid progenitor (MEDEP) cells [63], two different types of immortalized cell lines were established. Human iPS cell lines have been forced to express *TAL1*, an early hematopoiesis transcription factor [64]. Human iPS-*TAL1* cell-derived erythroid progenitor (HiDEP) cell lines have been generated through the transduction with a lentiviral vector with a tetracycline (Tet)-inducible expression system for the expression of HPV16 E6/E7. Likewise, human umbilical cord blood-derived erythroid progenitor (HUDEP) cell lines have been produced through the same lentiviral vector from CD34^+^ HSCs of umbilical cord blood [59]. Both cell types showed an effective differentiation in presence of erythropoietin (EPO) alone, producing a following upregulation in the hemoglobin synthesis. The hemoglobin produced in both cell types possessed similar oxygen binding activities compared to physiologically normal RBCs in vivo. A following gene expression analysis revealed a similar profile of erythroid-specific markers, such as GATA1, EKLF, GFI1B, TAL1 and EPO receptor (EPOR), in both cell lines compared with erythroid cells derived from umbilical cord blood cultured in vitro [59]. While authors did not perform a quantification of enucleation percentage, both cell line types were able to complete the maturation to reticulocytes reducing average cell size after differentiation compared to undifferentiated cells. Moreover, HiDEP showed a higher viability and enucleation efficiency than HUDEP. In summary, HiDEP/HUDEP cell line establishment has proven to be the first reproducible and robust immortalization method.

A recent study described the first erythroid immortalized cell line with up to 30% of enucleated cells following differentiation, which has been reported to be able to recapitulate normal adult erythropoiesis [60]. Authors developed the Bristol Erythroid Line—Adult (BEL-A) from transduction of bone marrow CD34^+^ with a lentiviral Tet-inducible HPV16 E6/E7 expression system able to proliferate continuously over 190 days. After 100 days, cultured cells were induced to differentiation, producing stable reticulocytes, which were identical to differentiated peripheral blood CD34^+^. Unlike previous immortalized cell lines [59, 65], BEL-As expressed similar surface markers compared with control adult erythroid cells and to synthesize exclusively *β*-globin with 99.3% of hemoglobin A (HbA). Moreover, a co-localization assay displayed the nuclear co-localization of myosin IIb and F-actin, known to play a key role in the formation of a contractile actin ring for nuclear extrusion [66–68]. Thus, the BEL-A cell line displayed a great potential and feasibility, laying the basis for further investigations in erythropoiesis.

Although the HPV *E6/E7* integration into host genome and viral protein expression are considered random events, the immortalization system relying on the transduction of HPV16 E6/E7 has proven to be the most effective [65]. Kurita et al*.* demonstrated how genetic modifications due to E6/E7 activity are strongly related to variations in immortalization, expansion, differentiation and enucleation efficiency [61]. The authors established 37 different bone marrow-derived erythroid progenitors (BMDEP) lines from a common cell source. Although all lines showed several chromosomal abnormalities, only 50% of those had altered karyotypes. All lines were induced to differentiate, but only three preserved proliferation ability upon differentiation. Moreover, the three lines also displayed highly similar surface marker expression patterns compared to normal erythroid cells and less than about 25% of enucleation efficiency. The study showed higher differentiation/enucleation efficiency in established cell lines at the earliest time point [61].

The efficiency of BEL-A immortalization, expansion and differentiation prompted the establishment of an immortalized cell line model from patients with HbE/*β*-thalassemia [69]. Previous cell models derived from iPSCs of thalassemic patients showed poor expansion levels [70, 71], either aberrant or incomplete erythroid differentiation as well as predominantly expression of fetal hemoglobin [72, 73]. A Siriraj Bristol beta-thalassemia/hemoglobin E (SIBBE) cell line has been generated from isolated peripheral blood CD34^+^ cells of a *β*-thalassemic patient transduced with a Tet-inducible HPV E6/E7 vector, as previously reported [60]. After over 100 days, SIBBEs have been induced to differentiate and mature. Cells preserved phenotype, morphology and size similar to BEL-A cell lines, while a large portion of differentiating cells lead to an effective erythropoiesis-related apoptosis with an enucleation rate about 10% [74]. The enucleation and terminal maturation efficiency was unchanged between early-passage and late-passage SIBBEs following prolonged expansion. Hemoglobin profiles resulted similar to that of *β*-thalassemic patients [69].

Daniels et al*.* proved the robust replicability of the lentiviral Tet-inducible HPV16-*E6/E7* expression system to the immortalization of bone marrow CD34^+^, by comparing canonic BEL-A immortalization (t0) with that at different following time points [16]. BM CD34^+^-derived BEL-A cell lines were established at four time points, i.e., t3, t5, t7 and t9, and maintained in culture over 200 days. All cell lines showed an expansion rate around t0. Despite the different starting erythroid populations, all cell lines display a final population of mainly proerythroblasts. The enucleation and differentiation efficiency was also comparable to that of t0 BEL-A [16]. Then, authors tried to reproduce immortalization protocols on peripheral and cord blood CD34^+^. BEL cord (BEL-C) and BEL peripheral blood (BEL-P) cell lines have been generated and induced to expand for over 180 days. Both cell populations were represented by mainly proerythroblasts with an expansion rate comparable to BEL-A cells. Moreover, the BEL-C/BEL-P differentiation potential, hemoglobin profile and morphology were unchanged compared to their respective primary cell cultures. Lastly, BEL-C/BEL-P cells were able to complete maturation with maximum enucleation rates of about 26% [16]. The data obtained demonstrated the solidity of this immortalization approach regardless of the starting cell source.

All HPV16 E6/E7-based immortalization systems rely on the necessity to silence oncoviral proteins with a Tet-inducible expression system. However, this approach provides, during maturation steps, a dramatic reduction of cell viability and proliferation, as well as enucleation efficiency [60, 69]. Based on this, a novel immortalized erythroid cell line with a constitutive HPV16 E6/E7 expression has recently been developed [75]. The cell line (Erythroid Line from Lund University [ELLU]) was able to differentiate upon culture into a differentiation medium. ELLUs were generated from transduced bone marrow CD34^+^, while being maintained for over 90 days. Ten different ELLU clones from the same cell source were established to evaluate hemoglobin synthesis and maturation/differentiation capacity. In all clones, the E6/E7 expression was paralleled with a significant reduction of p53 expression compared to HiDEP [59]. The expression of BCL11A, a key regulator for globin switching, has been exhibited in 9 clones out of 10. Moreover, consistent with BCL11A expression, ELLU hemoglobin profile was characterized by a higher conversion of $\gamma$-globin. Nevertheless, a lower *β*-globin expression resulted in loss of maturation and enucleation efficiency of ELLU cells. Only two clones that expressed a higher amount of *β*-globin were able to differentiate faster than others [75]. This investigation proved that the constitutive E6/E7 expression can allow the constitution of established cell lines with hemoglobin expression and enucleation rate comparable with those transduced with an inducible system.

Although several studies have demonstrated a high immortalization efficiency of CD34^+^ HPCs, their collection is too invasive and expensive. Thus, a recent study described the establishment of two immortalized erythroid progenitor cell (iEPC) lines with a novel culture protocol based on the transduction of peripheral blood mononuclear cells (PBMCs) with HPV16 E6/E7 (PBiEPC) [76]. PBiEPC-1 showed a shorter time of pre-immortalization after transduction compared to PBiEPC-2 line; the E6/E7 expression levels were inversely proportional to pre-immortalization time [61]. Using the same protocol, authors developed a CD34^+^iEPC cell line with a long pre-immortalization time, i.e., over 100 days, expressing limited amounts of E6/E7. Three cell lines have been induced to differentiate showing distinct differentiation time, enucleation rate and hemoglobin synthesis. PBiEPC-1s, immortalized at an earlier stage of erythropoiesis, showed a prolonged differentiation time, 18 days, with a high number of cultured cells and highest enucleation rate, estimated as ~ 20%. Contrariwise, PBiEPC-2s, immortalized at a later stage, displayed the lowest cell proliferation time, only 8 days, and enucleation rate, ranging 2–5%. After 13 days, CD34^+^ iEPCs completed their differentiation with an enucleation rate of ~ 15%. In all three cell lines, both *α*- and *β*-globin genes were upregulated compared to HUDEP, while CD34^+^ iEPC cell line showed a remarkably high level of *γ*-globin expression [76]. PBiEPC-1 represents the first immortalized erythroid cell line, derived from peripheral blood, with differentiation and enucleation capacities similar to bone marrow CD34^+^-derived immortalized cell lines.

As an alternative approach, lentiviral vectors containing sequences from different viral strains have been established. Wong et al*.* produced an immortalized CD36^+^ erythroblast (CD36E) cell line from CD36^+^ erythroid progenitor cells (EPCs), using a combination of three lentiviral vectors carrying Simian virus 40T-antigen (SV40T), *hTERT* and HPV16-*E6/E7* genes [65]. CD36^+^ EPCs increased their proliferation/expansion activity through the expression of HPV16-*E6/E7* alone and combined with *hTERT*, but not with SV40T, both alone and in combination. The immortalized cell line showed an increase of hemoglobin-producing cells, about 27% and a dramatic switching from HbA to HbF compared with CD36^+^ EPCs. Moreover, surface cell marker profile analysis highlighted the loss of hematopoietic stem cell phenotype with the absence of CD34. Inversely, the transforming activity of HPV16-*E6/E7* leads to chromosomal translocations and aneuploidy. Moreover, gene expression analysis displayed a significant increase of several lymphoid-relating factors and interferon regulatory factor 4, involved in proliferation activity of multiple myelomas, [77] and a decrease of erythroid differentiation-related factor, involved in hemoglobin assembly processes [78].

#### Controlled exogenous gene expression-based immortalization approaches

The ability of the erythroid cell reprogramming, by the controlled exogenous expression of specific genes, provides a relevant alternative to genetic modifications, which directly modify cell genome (Table 2) [59, 65]. Moreover, the controlled exogenous expression focuses the attention on novel genes involved in proliferative and self-renewal activities. Hirose et al*.* generated immortalized erythrocyte progenitor cells (imERYPCs) from hiPSC/hESC with self-replication potential with the transient upregulation of *c-MYC* and *BCL-XL*, which emulates physiological activity of immature erythroblasts proliferation [79]. In this study, HPCs, derived from hiPSC/hESC, have been transduced with a doxycycline (DOX)-inducible lentiviral vector including *c-MYC* and *BCL-XL*. The exogenous overexpression of these two proteins in the presence of EPO displayed increased proliferation and exponential growth for 6 months. Indeed, silencing both genes imERYPCs reduced cell growth, while cells showed significant modifications in morphology within 7 days, from immature erythroblasts to mature polychromatic/orthochromatic erythroblasts, in agreement with the canonic erythrocyte maturation [80, 81]. At day 7, imERYPCs showed an increase of hemoglobin synthesis comparable to that in peripheral blood RBCs in vitro. Moreover, *γ*-globin and HbF (*α*2*γ*2) levels in imERYPCs have been described as similar to that in cord blood-derived erythrocytes [79]. Upon *c-MYC* and *BCL-XL* silencing, gene expression analyses indicated an elevated expression of GATA1, RAF1 and endogenous BCL-XL and a down-regulation of GCN5 during maturation, similar to those in human erythrocyte equivalents. In particular, GATA1 and RAF1 are strongly involved in RBCs maturation and heme synthesis [82], while GCN5 down-regulation contributes to chromatin condensation [34]. Furthermore, significant results have been obtained from imERYPCs intraperitoneal injection into non-obese diabetic severe combined immunodeficiency mice. Cell lines strongly improved enucleation average, which has been estimated as > 90% at day 1 post-injection, underlining the need to complete erythroid maturation program in vivo [79].

**Table 2:** In vitro generation of immortalized red blood cell line using controlled exogenous gene expression-based approaches

Reference	Vector/plasmid/other immortalization systems for	Immortalization genes	Inducible	Cell origin	Cells used	Proliferation time	Hemoglobin production	Percent of enucleation
Hirose et al. [79]	Lentiviral vector	c-MYC or BCL-XL	DOX-inducible	Neonatal dermal fibroblasts,	ESCs/iPSCs	~ 6 months	*γ*- > *β*-globin	In vitro*:* 0.36%;
				PB				In vivo*:* > 90%
Huang et al. [83]	Retroviral vector	OCT4, SOX2, KLF4, c-MYC and shRNA antiTP53	No	UCB	ESCs	~ 3 months	*γ*- > *β*-globin	~ 30%
Geiler et al. [85]	PiggyBac transposon vector and a helper plasmid encoding the transposase	SPI-1	No	PB	CD34+ and CD34−	~ 1.5 months	–	–
Lee et al. [87]	Lentiviral vector	c-MYC or BCL-XL	No	PB	CD71 + CD235+	N.R	N.R	N.R
Couch et al. [89]	CRISPR/Cas9 system	Mutations into exon 17 of the KIT gene	No	–	HUDEP-2 cell line	8 months	Amount of *γ*-/*β*-globin-type mutation related	< 10%

BM, bone marrow; DOX, doxycycline; EPCs, erythroid progenitor cells; iPSCs, induced pluripotent stem cells; N.R., not reported but evaluated; PB, peripheral blood; UCB, umbilical cord blood; –, not reported and not evaluated

Huang et al*.* established immortalized-induced erythroblast (iE) cell lines through self-renewal reprogramming mediated by the so-called original Yamanaka reprogramming factors, which play an important role in stem cell induction [83]. Authors transfected cord blood mononuclear cells (CBMNCs) with retroviral vectors in order force the expression of five candidate factors for the self-renewal reprogramming, i.e*., OCT4*, *SOX2*, *KLF4*, *c-MYC* transgenes and a short hairpin RNA (shRNA) against human *TP53* gene [84]. The erythroid immature phenotype and morphology were maintained in near 100% of cells throughout the expansion period, extending growth rate and highest expansion potential for about 12 months. A similar expression level of SOX2, c-MYC and p53 compared to CB primary culture-expanded erythroblasts was found. Moreover, SOX2, c-MYC and p53shRNA have been reported as essential and sufficient factors for deriving iE cells. The global gene expression profile of iEs underlined a high similarity with primary CB erythroblasts compared to reprogrammed iPSC and ESC cells [83]. After the induction of differentiation, iEs cell diameter gradually decreased. Moreover, the hemoglobin produced and stored increased, although qPCR analysis underlined a dramatic upregulation of *γ*-globin/fetal hemoglobin. The erythroid terminal differentiation and enucleation efficiency, about 30% after 16 day, were comparable to those of primary culture-expanded CB erythroblasts [83]. The reprogramming efficiency of CB offers an interesting alternative of viral transforming factors in the establishment of functional immortalized erythroid cell lines.

The immortalization of a peripheral blood CD34 + HSC-derived erythroblast cell line has recently been described by using an innovative genetic engineering approach relying on the overexpression of SPI-1 [85]. This gene encodes for a transcription factor, which plays a crucial role in regulating the hematopoietic development [86]. In this study, HSC-derived erythroblasts have been transfected with a transposon vector construct containing an engineered SPI-1 under the control of a tetracycline-responsive promoter. Transfected erythroblasts proliferated for 45 days preserving an undifferentiated phenotype (95.9% of CD117^+^CD71^+^ cells) similar to early proerythroblast [85]. Although promising data were obtained with this approach, differentiation and enucleation rates have not been evaluated.

The generation of different immortalized erythroid cell lines led researchers to optimize cell lines growth conditions and to reduce the production costs. An optimal erythroid expansion requires hormones, factors and cytokines, as medium supplements, which significantly increase in production costs. An in-house medium has recently been developed using a design-of-experiment (DOA)-based optimization of the peripheral blood CD71^+^CD235a^+^-immortalized erythroblasts (ImEry) expansion [87]. The immortalization was performed through transduction with a lentiviral vector containing the human *c-MYC* and *BCL-XL* genes. Upon transduction, ImEry cells showed expression of important erythroid progenitor markers. The optimized medium resulted threefold more cost-effective, but also induced a ~ 75% increase in ImEry cells expansion. However, ImEry enucleation and terminal maturation rates were drastically low. The study clarified which factors, among those commonly used, can be considered essential for a correct erythroid expansion [88]. In addition, the medium depletion of L-serine, L-cystine and L-methionine induced a drastic decrease in ImEry cell expansion, which is improved with their over-supplementation.

The clustered regularly interspaced short palindromic repeats (CRISPR)/Cas9 system has recently been applied for immortalizing erythroid cells [89]. In particular, the HUDEP-2 cell line [59] was transfected with a CRISPR/Cas9 and sgRNA KIT-targeting vector in order to introduce mutations in exon 17 of the *c-Kit* proto-oncogene. Genetic modifications occurred in 16 clones allowed the c-Kit gene, encoding for the stem cell factor receptor (SCFR), to be constitutively activated [90, 91]. All KIT with constitutively activating transformation (CAT) cell clones were able to be independent from SCF, a factor implicated in erythroid cell survival, expansion and differentiation. A fraction of clones unexpectedly showed complete or partial independence from EPO. The KITCAT cell phenotype, as early erythroblast, resulted similar to that of the parental HUDEP-2 line, although KITCATs resulted significantly larger in both cell diameter and nucleus. Similarly to HUDEP-2 cell line, KITCATs presented a slower maturation compared to in vivo erythroblasts. Nevertheless, KITCAT cell lines displayed a higher expansion than HUDEP-2 cells [89]. Lastly, both HUDEP-2 cells and KITCATs demonstrated a similar enucleation rate, about < 10%, probably due to a constitutive KIT activity that could have partially inhibited enucleation. To summarize, the cell line established displayed SCF-independent expansion and maturation, reducing, in turn, culture costs by approximately half.

## Conclusions and future perspectives

Red blood cell transfusion is currently the most used therapeutic approach in clinical practice. The increasingly high demand compared to the limited supply of transfusable blood has led researchers to develop new approaches for the in vitro production of erythroid cells. The establishment of immortalized erythroid cell lines provides a viable alternative over donor blood, which is commonly associated with potential risk factors, such as bloodborne viral infections, alloimmunization and transfusion-related complications [92]. The longest proliferation time and the highest survival capacity of immortalized cell lines allow the generation of a large number of cells over time, which can differentiate. Most of the studies performed this step through the inducible/constitutive expression of HPV16 E6/E7. This system shows a considerable efficiency of cell immortalization, as well as remarkable selectivity for proerythroblasts and early basophilic erythroblasts [16]. Moreover, the early-stage immortalization is commonly linked to an increase of differentiation time, as well as cell viability and enucleation rate [76]. On the contrary, the oncogenic activity of HPV proteins has often been associated with an increase of genomic instability, chromosomal abnormalities and altered karyotypes [61, 62, 65, 93], which is observed in transformed/tumor cells [94, 95]. The SV40 erythroid immortalization potential is still largely unknown. Indeed, it is currently known that human cells display a higher resistance to SV40 lytic cycle. Furthermore, the lower permissiveness to virus itself might impair the cell immortalization process [96]. Several studies proved SV40 immortalization activity in different cell types such as fibroblasts and B/T lymphocytes [97–99]. An increase in normal human B-lymphocytes lifespan following transfection with an SV40-Tag vector has been reported [100]. Further studies are required to evaluate the feasibility and effectiveness of this approach in erythroid cell immortalization.

Most of studies establishing erythroblast cell lines have been reported to allow cell proliferation in vitro for averagely 100 days. In addition, cells preserved their immortalized property and progenitor erythroid morphology during the entire proliferation time until differentiation induction. Nevertheless, it is clear that the prolonged maintaining of an immortalized cell line, which is necessary to increase the number of cultured cells, is largely connected to a considerable usage of culture medium, as well as medium supplements. Although some studies have been able to limit significantly medium costs [87, 89], the culture medium economic impact, particularly for essential medium supplements such as SCF, EPO, interleukin 3 and serum, still remains an unresolved challenge to be further explored. The development of engineered immortalized cell lines able to be independent from some of these growth/maintaining factors offers an appealing solution for culture costs reduction.

The average enucleation rate in previous studies did not exceed 40%. Enucleation is a complex multistep process characterized by several cellular and molecular mechanisms finely regulated [25]. Endosomal vacuoles formation, mitochondrial aggregation as well as chromatin condensation are crucial steps in erythroid maturation/enucleation, which have been effectively replicated in vitro. On the contrary, it is noted as some important events, such as erythroid–macrophage peptide (EMP) interactions, are implicated in macrophage direct induction of enucleation in vivo [101–103]. The addition of molecules able to mimic macrophage peptides could be a prominent approach that deserve further attention [92]. Moreover, the supplementation of proteins involved in autophagy/apoptosis as well as inhibitors/activators of enzymes implicated in histone post-translational modifications [32, 33] could increase the enucleation efficiency. In addition, the development of more efficient filtration and/or new non-enucleated cell elimination systems is urgently required.

The large-scale ex vivo production of red blood cells still remains a barrier to overcome. In recent years, various studies achieved important advancements in the production yield of erythroid cultures with the design of bioreactors that allow three-dimensional cell growth [104, 105]. Heshusius et al*.* developed a good manufacturing practice (GMP) culture medium adapted to a culture protocol, which led to the production of mature erythroblasts with > 90% enucleation. The bioreactor system was able to induce a 3 × 10^7^-fold increase of the production of pure erythroid cultures, using PBMCs without prior CD34^+^ isolation [106]. In order to implement an efficient, cost-effective and safe procedure, a whole range of issues should be solved, including culture medium and medium supplement replacement, exclusion of expensive/elaborate growth/differentiation protocols and more selective and frequent safety control introduction of cell cultures [107]. The use of co-culture of xenogeneic stromal lines, such as OP9 and MS5, proved a remarkable increase of maturation and cell population expansion. However, the high number of stromal cells needed, the complexity of the employed techniques/protocols alongside the considerable risk of contamination by xenogeneic pathogens still make this option unfeasible [107, 108]. During production processes, several controls are required to monitor multiple physical and biochemical factors of immortalized erythroid cell culture, such as enucleation and globin transition efficiency and possible genotype alterations. Although several studies have established immortalized erythroid cell lines with either partial or exclusive production of *β*-globin [60, 75], the enucleation efficiency still remains a considerable limit to large-scale blood production. The large-scale production of immortalized erythroid cells should be further implemented.

In conclusion, a large-scale, cost-effective production of blood cells is not yet a routine clinical procedure as depending on the still ongoing optimization of the erythroid culture conditions [109]. The in vitro differentiation and terminal maturation remain currently less efficient compared to that obtained in vivo. The highest survival capacity alongside the stable and longest proliferation time of immortalized erythroid cells could allow the generation of large number of cells over time, which are able to differentiate into red blood cells. In future, erythroid cells might represent a reliable and cost-effective method for a large-scale generation of blood cells.

## Data Availability

Data sharing is not applicable to this article as no new data were created or analyzed in this study.

## Abbreviations

WHO: World Health Organization
RBCs: Red blood cells
HSPCs: Hematopoietic stem and progenitor cells
CMP: Common myeloid progenitor
BFU-E: Burst-forming unit erythroid
CFU-E: Colony-forming unit erythroid
HSCs: Hematopoietic stem cells
BM: Bone marrow
PB: Peripheral blood
UCB/CB: Umbilical cord blood/cord blood
G-CSF: Granulocyte colony-stimulating factor
hPSCs: Human pluripotent stem cells
hESCs: Human embryonic stem cells
iPSCs: Induced pluripotent stem cells
HPV: Human papillomavirus
hTERT: Human telomerase reverse transcriptase
HiDEP: Human iPS-TAL1 cell-derived erythroid progenitor
Tet: Tetracycline
HUDEP: Human umbilical cord blood-derived erythroid progenitor
EPO: Erythropoietin
EPOR: Erythropoietin receptor
BEL-A/P/C: Bristol erythroid line adult/peripheral blood/cord
Hb: Hemoglobin
BMDEPs: Bone marrow-derived erythroid progenitors
SIBBE: Siriraj Bristol beta-thalassemia/hemoglobin E
ELLU: Erythroid Line from Lund University
iEPCs: Immortalized erythroid progenitor cells
ImERYPCs: Immortalized erythrocyte progenitor cells
iEs: Immortalized-induced erythroblasts
CBMNCs: Cord blood mononuclear cells
shRNA: Short hairpin RNA
DOA: Design of experiment
ImERYs: Immortalized erythroblast cell line
CRISPR: Clustered regularly interspaced short palindromic repeats
SCF: Stem cell factor
KITCATs: KIT with constitutively activating transformation cell line
EMP: Erythroid macrophage peptide
